# Risk for Rabies Importation from North Africa

**DOI:** 10.3201/eid1712.110300

**Published:** 2011-12

**Authors:** Philippe Gautret, Florence Ribadeau-Dumas, Philippe Parola, Philippe Brouqui, Hervé Bourhy

**Affiliations:** Author affiliations: Hôpital Nord, Marseille, France (P. Gautret, P. Parola, P. Brouqui);; Institut Pasteur, Paris, France (F. Ribadeau-Dumas, H. Bourhy)

**Keywords:** Rabies, North Africa, Europe, viruses, importation, travelers, zoonoses

## MEDSCAPE CME

Medscape, LLC is pleased to provide online continuing medical education (CME) for this journal article, allowing clinicians the opportunity to earn CME credit.

This activity has been planned and implemented in accordance with the Essential Areas and policies of the Accreditation Council for Continuing Medical Education through the joint sponsorship of Medscape, LLC and Emerging Infectious Diseases. Medscape, LLC is accredited by the ACCME to provide continuing medical education for physicians.

Medscape, LLC designates this Journal-based CME activity for a maximum of 1 *AMA PRA Category 1 Credit(s)*^TM^. Physicians should claim only the credit commensurate with the extent of their participation in the activity.

All other clinicians completing this activity will be issued a certificate of participation. To participate in this journal CME activity: (1) review the learning objectives and author disclosures; (2) study the education content; (3) take the post-test with a 70% minimum passing score and complete the evaluation at www.medscape.org/journal/eid; (4) view/print certificate.

**Release date: November 22, 2011; Expiration date: November 21, 2012**

## Learning Objectives

Upon completion of this activity, participants will be able to:

- Distinguish the animal associated with most cases of fatal rabies
- Evaluate the epidemiology of rabies in North Africa
- Assess how rabies cases are managed in France
- Analyze who should receive strong consideration for preexposure vaccination against rabies prior to travel to North Africa

## Editor

**P. Lynne Stockton, VMD, MS, ELS(D),** Technical Writer/Editor, *Emerging Infectious Diseases. Disclosure: P. Lynne Stockton, VMD, MS, ELS(D), has disclosed no relevant financial relationships.*

## CME AUTHOR

**Charles P. Vega, MD**, Associate Professor; Residency Director, Department of Family Medicine, University of California, Irvine. *Disclosure: Charles P. Vega, MD, has disclosed no relevant financial relationships.*

## AUTHORS

*Disclosures:* ***Philippe Gautret, MD; Florence Ribadeau-Dumas, MD; Philippe Parola, MD, PhD;*** *and* ***Philippe Brouqui, MD, PhD,*** *have disclosed no relevant financial relationships.* ***Hervé Bourhy, MD,*** *has disclosed the following relevant financial relationships: served as a speaker or a member or a speakers bureau for Sanofi Pasteur.*
