# Supplementary material for: Risk for Rabies Importation from North Africa
Source: Emerg Infect Dis. 2011 Dec;17(12):2187–93. doi: 10.3201/eid1712.110300 (PMC3311213; doi:10.3201/eid1712.110300)
Supplement: Related texts

# Review article: Risk for Rabies Importation from North Africa

**DOI:** 10.3201/eid1712.110300

**Authors:** Philippe Gautret, Florence Ribadeau-Dumas, Philippe Parola, Philippe Brouqui, Hervé Bourhy

**Affiliations:** Author affiliations: Hôpital Nord, Marseille, France (P. Gautret, P. Parola, P. Brouqui);; Institut Pasteur, Paris, France (F. Ribadeau-Dumas, H. Bourhy)

## Abstract

A retrospective study conducted in France indicated that a large proportion of patients injured by potentially rabid animals while in North Africa did not seek pretravel advice, and some had not received proper rabies postexposure prophylaxis while in North Africa. As a result, imported human rabies cases are still being reported, and the need for postexposure prophylaxis after exposure in North Africa is not declining. Tourists are generally unaware of the danger of importing potentially rabid animals and of the rules governing the movement of pets. In France, for example, rabid dogs have frequently been imported from Morocco to France through Spain. This situation imposes heavy social and economic costs and impedes rabies control in Europe. Rabies surveillance and control should therefore be reinforced in North Africa, and travelers to North Africa should receive appropriate information about rabies risk and prevention.

Among all human deaths from rabies, >99% occur in the developing world and result from bites from rabid dogs (*1*). Although effective and economic control measures are available, rabies remains a neglected disease in most of these countries (*2*). Animal-associated injuries in travelers to rabies-endemic countries are not infrequent (*3*) and pose a serious health threat to persons visiting such areas (*4*). Rabies is a serious public health concern in North Africa (Morocco, Algeria, Tunisia, Libya, Egypt, and Sudan, as defined by the United Nations), causing heavy social and economic costs. The possibility of its reintroduction threatens western European countries, where terrestrial carnivores are presently rabies free (*5**,**6*).

## Rabies in North Africa

Although rabies is endemic to North Africa (*7*), accurate data on incidence are scarce (*8*), and better surveillance is needed. Initiatives have recently been developed to share information and experiences, provide reliable rabies epidemiologic data, raise rabies awareness, improve access to prevention, and design programs for rabies control. These initiatives include the Africa Rabies Expert Bureau (www.afroreb.info), the global multidisciplinary European Union–funded RABMED CONTROL project (www.rabmedcontrol.org), and the Global Alliance for Rabies Control (www.rabiescontrol.net). Some of these initiatives are expected to provide detailed epidemiologic data about the rabies situation in North African countries. Although rabies incidence in North Africa is certainly underestimated (*2**,**8**,**9*), according to the most recent reports in the World Health Organization (WHO) RabNet database (www.who.int/globalatlas/default.asp), RABMED CONTROL data, and other sources (*5**,**9**–**12*), many human rabies cases are regularly reported in North Africa. The annual incidence varies from 0.02 cases/100,000 population in Tunisia to 0.1 cases/100,000 population in Egypt (Table 1). These disparities may result from differences in the epidemiologic status of animal rabies. More likely, however, they reflect the combined effects of variations in the reliability of epidemiologic data, the implementation of primary wound care, and the accessibility of rabies postexposure prophylaxis (PEP). Indeed, rabies PEP accessibility is subject to large disparities; the lowest rates (0.4 persons receiving rabies PEP/1,000 population) are reported in Sudan, and the highest (3.3 persons receiving rabies PEP/1,000 population) are reported in Tunisia (Table 1).

**Table 1:** Reported rabies cases in humans and animals, North Africa, 2000–2009*

Country	Annual no. human deaths/100,000 population	Annual no. rabies PEP in humans/100,000 population	No. rabies cases in animals (years)
Algeria	0.06†	2.2†	2,206 (2000–2008), 754 (2009)
Egypt	0.1‡	1.9§	5 (2000, 2006, and 2007)¶
Morocco	0.07†	1.6†	3,600 (2000–2008, including 343 in 2007)
Sudan	0.04†	0.4‡	101 (2000–2007, including 38 in 2007)
Tunisia	0.02†	3.3†	1,253 (2000–2007, including 102 in 2007)

*PEP, postexposure prophylaxis. Data sources include World Health Organization RabNet database (www.who.int/globalatlas/default.asp) and RABMEDCONTROL (www.rabmedcontrol.org) (*5**,**9**–**12*). †Calculated for 2000–2007. ‡Calculated for 2007. §Calculated for 2000. ¶No data available for other years.

In Morocco, 90% of human cases of rabies are caused by dog bites. Cases occur mainly in rural areas in Kenitra, Casablanca, and El Jadida (*10*). In Algeria, human deaths from rabies occur mostly in the coastal and northern part of the country (*11*). In Tunisia, human cases are localized in the northern (Bizerte and Béja), central and eastern rural (Siliana, Kairouan, Kasserine, Sidi Bouzid, and Gafsa), and southern coastal (Gabes and Medenine) parts of the country.

Most reported cases of rabies in animals are in dogs, which account for >40% of animals confirmed rabid by laboratory investigations. Rabies cases are also reported for cats, cattle, goats, sheep, and horses. However, data collected for animal rabies (Table 1) are only partial and do not represent the true status of this infection in the field (*8*). In Tunisia, mass vaccination campaigns directed at the canine population, together with the elimination of free-roaming dogs, has proven somewhat effective (*13*). However, rabies in dogs is still prevalent in Algeria, Morocco, and Tunisia (Table 1) because of ineffective implementation of rabies control and vaccination programs for these reservoir animals (*5**,**8**,**14**,**15*).

The information available from Egypt is clearly incomplete (*16*). Elimination of free-roaming cats and dogs has been conducted in Egypt with little effect (*5*). In Sudan, animal rabies is reported mainly in dogs but also in goats and donkeys (*17**,**18*). From Libya, no information about rabies in humans or animals has been available for many years; the country has declared itself free of canine rabies, although rabies is present in all neighboring countries (*19*). This description of the current rabies situation in North Africa is based on reported cases only and does not necessarily represent the real epidemiologic situation in the area, given the underreporting that results from nonexhaustive surveillance.

## Risk for Importing Human Rabies from North Africa to Europe

North Africa is a popular tourist destination; in 2005, it accounted for 12% of all international travel from France. The most popular destinations were Morocco, Tunisia, and Egypt; these countries together accounted for 89% of travel to North Africa, with 950,000, 920,000, and 590,000 visits, respectively. Algeria accounted for 5% of travel to North Africa, with 140,000 visits. No data are available for Sudan or Libya (*20*). Persons who emigrated from North Africa to France also travel frequently to their countries of origin to visit friends and relatives. This travel pattern also creates a pathway for rabies reintroduction to France. During the first half of the 20th century, canine rabies progressively disappeared from most countries in western and southern Europe (*21*). At the end of the 1940s, the epizootic of vulpine rabies spread from Poland into the rest of Europe (*22*). Today, oral vaccination of foxes has pushed vulpine rabies back into eastern Europe (*23*). Maintaining rabies-free status among terrestrial carnivores, however, incurs considerable costs. Meanwhile, the legal and illegal importation of live animals imposes a continual risk for reimportation, as does the lack of awareness by travelers visiting rabies-endemic areas (*6**,**24*).

In France, primary health care for patients seeking rabies PEP is delivered through an official network of antirabies medical centers (ARMCs), which facilitate the accurate epidemiologic evaluation of animal-related injuries for which patients require rabies PEP. Since 1970, a total of 21 human deaths from rabies have been recorded in France; these cases resulted in a large number of rabies PEP treatments for contacts (Table 2). Among these fatal cases, 19 were imported (50% originated in North Africa, mainly in Algeria and Morocco), 1 was acquired in French Guiana, and 1 was acquired through a cornea transplant from a donor infected in Egypt. During the past decade, 2 cases were acquired in Morocco by travelers from Austria and Germany (*26**,**27*). Other cases, imported to the United Kingdom, Germany, Sweden, and Finland, were acquired from India, Thailand, the Philippines, Nigeria, and South Africa (*28**–**31*). These data reflect the relevance of specific patterns of international travel between European and rabies-enzootic countries.

**Table 2:** Cases of rabies in humans, France, 1970–2010*

Year of death	Patient age, y/sex	Country of exposure	Animal species	Incubation time	Time between illness onset and death	No. contacts who received rabies PEP
1970	3/M	Niger	Cat	10 d	9 d	Unknown
1973	10/M	Gabon	Dog	11 mo or 15 d	20 d	Unknown
1976	5/M	Gabon	Dog	45 d	1 mo	Unknown
1976	18/M	Algeria	Dog	Unknown	23 d	1
1976	28/M	Morocco	Unknown	Unknown	1 mo	Unknown
1976	10/M	Algeria	Dog	1 mo	18 d	Unknown
1977	2/M	Gabon	Dog	18 d	1 d	5
1977	4/M	Morocco	Dog	1 mo	2 d	25
1979	57/F	Egypt	Dog	2 mo	10 d	12
1979	36/M	Egypt	Human (cornea transplant)	1 mo	15 d	128
1980	4/M	Tunisia	Dog	2.5 mo	3 d	66
1982	40/M	Senegal	Dog	122 d	30 d	Unknown
1990	28/M	Mexico	Dog	47 d	10 d	1
1992	3/M	Algeria	Dog	1 mo	3 wk	143
1994	46/M	Mali	Dog	3 mo	11 d	36
1996	3/M	Madagascar	Dog	2 mo	6 d	290
1996	60/M	Algeria	Dog	2 mo	5 d	45
1996	71/M	Algeria	Dog	40 d	3 d	35
1997	50/F	India	Dog	12 d	56 d	36
2003	3/M	Gabon	Dog	>2 mo	10 d	142
2008	42/M	France (French Guiana)	Bat	Unknown	16 d	90

*Adapted from Peigue-Lafeville et al. (*25*). PEP, postexposure prophylaxis.

Of the 133,852 patients who consulted an ARMC in France during 1996–2009, a total of 6.7% of exposures to animals suspected of having rabies occurred while the patient was traveling outside France (French National Rabies Reference Centre, unpub. data). Persons who acquired animal-related injuries abroad accounted for 11.2% of patients who received rabies PEP during the same period, a figure that rose from 6.8% in 1996 to 17.8% in 2009 (Figure). Among treated patients who were injured abroad, 29.9% had returned from North Africa. This figure underscores the role of North Africa in rabies PEP epidemiology in France (Table 3). Need for rabies PEP seems to be particularly high after travel to Algeria. In 2005, only 5% of international tourists from France visited Algeria, but persons injured in Algeria accounted for 21.4% of patients seeking care in France after possible rabies exposure while abroad. Furthermore, 25% of all human rabies cases observed in mainland France since 1970 resulted from exposure in Algeria. In France, 94.7% of patients injured in North Africa received rabies PEP (Table 3, Table 4), compared with 53% of those injured elsewhere, and 16.1% of rabies PEP given included rabies immunoglobulin, compared with only 5.8% given to persons injured elsewhere. This higher rate of rabies PEP among travelers injured in North Africa reflects what is understood about rabies epidemiology in the region and the perception of the risk by physicians, which is largely influenced by the number of rabies infections that developed in France in persons exposed while in North Africa. Children are especially at risk. Since 1970, children <15 years of age have accounted for 50% of human rabies cases in mainland France; among these, 8 of 10 cases have occurred in children <6 years of age. Moreover, the proportion of children <15 years of age receiving rabies PEP in France is higher when their rabies exposure occurred in North Africa (Table 4). Regional variations also exist within France. In a recent study conducted among ≈424 international travelers seeking care for animal-associated injuries at the ARMC in Marseille, southern France, most (41.5%) had traveled to North Africa, and most injuries were from dogs and cats. A correlation was found between the country of exposure and the implicated species; in Algeria, the most implicated animals were dogs and in Tunisia, cats (*32*). During 2004–2010, among 90 patients exposed in North Africa and seeking care in Marseille, 53 (59%) had traveled for tourism and 35 (39%) had visited friends and relatives; however, 26 (69%) travelers injured in Algeria had visited friends and relatives (Table 5). The French-speaking North African community is particularly large in Marseille, where it accounts for one third of the total immigrant population.

**Figure:** Proportion of patients injured in countries in North Africa compared with those injured in countries in other foreign regions among all patients who consulted an antirabies medical center and received rabies postexposure prophylaxis (PEP), France, 1996–2009.

**Table 3:** Patients possibly exposed to rabies while visiting North Africa, 1996–2009*

Country of exposure	No. exposed patients visiting ARMC	No. patients receiving rabies PEP	Proportion of all foreign exposures occurring in country, %
Algeria	649	603	7.6
Egypt	117	103	1.3
Libya	10	9	0.1
Morocco	1,036	992	12.5
Sudan	8	8	0.1
Tunisia	687	659	8.3
Total	2,507	2,374	29.9

*Original data from the French National Reference Centre on rabies. ARMC, antirabies medical center; PEP, postexposure prophylaxis.

**Table 4:** Patients receiving rabies PEP among patients consulting an antirabies medical center, France, 1996–2009*

Location of possible exposure	% Patients age <15 y among those who received rabies PEP†	% Patients age <6 y among those who received rabies PEP†	% Patients who received rabies PEP among those age <15 y†	% Patients who received rabies PEP among those age <6 y†	Total no. patients‡		Total no. patients who received rabies PEP
					<15 y of age	<6 y of age	
North Africa	29.4	12.3	94.8	95.1	735	306	2,374
All other	24.2	8.8	44.1	37.9	37,895	16,093	69,145

*Original data from the French National Reference Centre for Rabies. PEP, postexposure prophylaxis. †p<10^–6^. ‡Regardless of rabies PEP status.

**Table 5:** Reasons for travel among 90 patients injured in North Africa and consulting an antirabies medical center, Marseille, France, 2004–2010

Reason for travel	Place of exposure, no. (%) patients				
	Algeria	Morocco	Tunisia	Egypt	Total
Tourism	10 (27.8)	24 (72.7)	17 (89.6)	2 (100)	53 (58.9)
Visit with friends and relatives	26 (72.2)	8 (24.3)	1 (5.2)	0	35 (38.9)
Business	0	1 (3.0)	1 (5.2)	0	2 (2.2)
Total	36	33	19	2	90

Generally, only a few travelers to North Africa seek pretravel advice before departing, which is not surprising because specific vaccination and malaria prophylaxis are not required before travel to North Africa. In 2009 in the Paris ARMC, only 1 (1.2%) of 82 travelers from France who had been injured by animals while in North Africa had been vaccinated before traveling (Paris ARMC, unpub. data). Knowledge about rabies risk and preventive measures among persons traveling from France to rabies-endemic countries is limited (*33*).

## Importation of Rabid Animals

Rabid animals have been repeatedly imported by travelers into France; most animals originated in Morocco and were transported through Spain by car (*34**–**37*). During 2000–2009, a total of 8 imported rabies cases were reported. All infected animals were dogs imported from rabies-enzootic countries: 7 from Morocco and 1 from Gambia. Additionally, a dog that died in February 2008 had never traveled out of France. Epidemiologic investigations and molecular typing of the virus confirmed that this case was indirectly linked to an imported dog from Morocco that had died in late 2007; the imported dog had not been examined for rabies. No secondary transmissions to humans resulted from these animal cases. However, the identification of contacts at risk to ensure that they received appropriate care is costly and time-consuming (*6*). The sanitary regulation regarding rabies vaccination of all carnivores entering the European Union is essential for rabies control and must be strictly applied in areas of Europe that have been declared rabies free (*38*). This precaution applies to France in particular because the illegal pet importation route from Morocco through Spain to France is well known.

## Availability of Rabies Vaccine and Immune Globulin in North Africa

Modern imported cell culture vaccines are available in Morocco, Egypt, Sudan, and Tunisia. No data are available from Libya. Algeria has been using mouse brain rabies vaccine made in Algeria and is starting to use the cell culture vaccine; however, according to the Paris ARMC (in charge of 16.6% of patients receiving rabies PEP in France and 47.4% of patients exposed abroad in 2009 and receiving rabies PEP), 14 (53.8%) of the 26 patients who began their rabies PEP in Algeria still received mouse brain rabies vaccine in 2009. Equine rabies immunoglobulin is available in most prevention centers in Algeria, Morocco, and Tunisia (≈100 centers in Algeria, 147 in Morocco, and 206 in Tunisia) (*9*), but human rabies immunoglobulin is less widely available. Data on the availability of rabies immunoglobulin in Libya and Sudan are not available. However, a substantial proportion of travelers injured in North African countries do not receive adequate rabies PEP. Thus, in the Paris ARMC in 2009, of 32 patients requiring rabies immunoglobulin (patients with grade III exposure who had never previously received preexposure or postexposure rabies vaccine) and whose PEP was started in North Africa, 18 (56.3%) neither received rabies immunoglobulin locally nor consulted with a doctor when they returned to France in time to receive it. Rabies immunoglobulin must be administered <7 days after administration of the first vaccine dose (*39*). In contrast, of 9 (22.2%) patients with grade II exposure and no exposure to bats, 2 received rabies immunoglobulin in Algeria, although this treatment is not recommended by WHO guidelines (*1*). Such deviations were not observed among 26 patients returning from Morocco, Tunisia, or Egypt.

Similarly, in the Marseille ARMC during 1987–2005, among the 34 patients requiring rabies immunoglobulin whose PEP was started in North Africa, only 2 (5.9%) travelers received rabies immunoglobulin in the country of exposure and 23 (67.7%) travelers who received only vaccination in the country of exposure came to home clinics >7 days after receiving the first vaccine injection. After 7 days, administration of rabies immunoglobulin is useless and might even have a negative influence on active immune response (*40*).

## Conclusions

We recommend that rabies control measures and, in particular, rabies control and vaccination programs in dogs be further implemented in North African countries. Meanwhile, the persistent risk for rabies in these countries exists for travelers, as shown for France. A large proportion of travelers exposed to potentially rabid animals in North Africa did not seek pretravel advice from travel clinics, thus missing the opportunity to learn about rabies risks and preventive measures. Furthermore, many of these patients did not receive rabies PEP and rabies immunoglobulin in accordance with WHO recommendations in the country of exposure. This problem reinforces the need to inform and train health professionals in these countries with regard to recommended practice for rabies prevention for humans.

Finally, rabies control in Europe is impeded by lack of tourist awareness of the threat of importing rabies from countries where it is enzootic and of the rules governing movement of pets. The sanitary regulations regarding rabies vaccination of all carnivores entering the European Union (*38*) are essential for rabies control and must be strictly applied in European areas that have been declared free of rabies in terrestrial carnivores. These regulations define the requirements that dogs and cats must meet before entry into the European Union, with the aim of preventing entry of an infected but asymptomatic dog or cat from outside Europe.

We suggest that all travelers to North Africa be fully informed about rabies risk, adequate preventive measures, and risk of importing animals. For persons traveling to North Africa to visit relatives and who are at high risk for exposure to potentially rabid animals, rabies preexposure vaccination should be discussed; this precaution is especially wise for those undergoing repeated or long visits to places with no modern culture cell rabies vaccine and for groups at risk, such as children <15 years of age. As long as these measures and information are not properly implemented, costly capacities for surveillance of rabies in animals and humans, as well as for monitoring rabies exposures in humans, should be maintained.

## References

[1] World Health Organization. Rabies vaccines: WHO position paper—recommendations. Vaccine. 2010;28:7140–2. Epub 2010 Sep 8. 10.1016/j.vaccine.2010.08.08220831913

[2] Knobel DL, Cleaveland S, Coleman PG, Fevre EM, Meltzer MI, Miranda ME, Re-evaluating the burden of rabies in Africa and Asia. Bull World Health Organ. 2005;83:360–8.15976877PMC2626230

[3] Gautret P, Schwartz E, Shaw M, Soula G, Gazin P, Delmont J, Animal-associated injuries and related diseases among returned travelers: a review of the GeoSentinel Surveillance Network. Vaccine. 2007;25:2656–63. 10.1016/j.vaccine.2006.12.03417234310

[4] Meslin FX. Rabies as a traveler’s risk, especially in high-endemicity areas. J Travel Med. 2005;12:S30–40. 10.2310/7060.2005.1205516225804

[5] Barrat J. La rage dans le Bassin Méditerranéen. AFSSA Bull Epid. 2005;17:S5–6 [cited 2010 May 1]. http://agriculture.gouv.fr/IMG/pdf/be17_suppltrage.pdf

[6] Lardon Z, Watier L, Brunet A, Bernède C, Goudal M, Dacheux L, Imported episodic rabies increases patient demand for and physician delivery of antirabies prophylaxis. PLoS Negl Trop Dis. 2010;4:e723. 10.1371/journal.pntd.000072320582307PMC2889823

[7] Talbi C, Lemey P, Suchard MA, Abdelatif E, Elharrak M, Jalal N, Phylodynamics and human-mediated dispersal of a zoonotic virus. PLoS Pathog. 2010;6:e1001166. 10.1371/journal.ppat.100116621060816PMC2965766

[8] Dodet B. Fighting rabies in Africa: the Africa Rabies Expert Bureau (AfroREB). Vaccine. 2008;26:6295–8. 10.1016/j.vaccine.2008.04.08718617294

[9] Dodet B. The Africa Rabies Expert Bureau (AfroREB).The fight against rabies in Africa: from recognition to action. Vaccine. 2009;27:5027–32. 10.1016/j.vaccine.2009.06.03019560430

[10] Faouzi A, Anga L, Barkia A, Nenmamoune A, Amarouch H, Nourli J. La rage humaine au Maroc de 2000 à 2008. Rev Epidemiol Sante Publique. 2009;57:S27. 10.1016/j.respe.2009.02.093

[11] Metallaoui A. Rage: historique et situation épidémiologique en Algérie. Projet GCP/RAB/002/FRA. 2009 [cited 2010 May 1]. ftp://ftp.fao.org/docrep/fao/012/ak149f/ak149f00.pdf

[12] El Ghoul. Historique et situation épidémiologique en Tunisie. Projet GCP/RAB/002/FRA. 2009 [cited 2010 May 1]. ftp://ftp.fao.org/docrep/fao/012/ak150f/ak150f00.pdf

[13] Touihri L, Zaouia I, Elhili K, Dellagi K, Bahloul C. Evaluation of mass vaccination campaign coverage against rabies in dogs in Tunisia. Zoonoses Public Health. 2011;58:110–8. 10.1111/j.1863-2378.2009.01306.x20042063

[14] Artois M, Ben Osman F, Kilani M, Wandeler A. Contribution nouvelle à la connaissance de l’écologie du chien en Tunisie. In: La rage et la brucellose dans les pays Méditerranéens et la Péninsule Arabe. Collection Fondation Marcel Mérieux; 1986. pp. 171–8.

[15] Matter H. Etude écologique d’une population canine. Maghreb Vétérinaire. 1987;312:65–8.

[16] Matter H, Blancou J, Benelmouffok A, Hammami S, Fassi-Fehri N. Rabies in North Africa and Malta. In: King AA, Fooks AR, Aubert M, Wandeler AI, editors. Historical perspective of rabies in Europe and the Mediterranean Basin. Paris and Geneva: World Organisation for Animal Health and World Health Organization; 2004. p. 185–99.

[17] Marston DA, McElhinney LM, Ali YH, Intisar KS, Ho SM, Freuling C, Phylogenetic analysis of rabies viruses from Sudan provides evidence of a viral clade with a unique molecular signature. Virus Res. 2009;145:244–50. Epub 2009 Jul 21. 10.1016/j.virusres.2009.07.01019628013

[18] Ali YH, Intisar KS, Wegdan HA, Ali EB. Epidemiology of rabies in Sudan. Journal of Animal Veterinary Advances. 2006;5:266–70.

[19] Burki T. The global fight against rabies. Lancet. 2008;372:1135–6. 10.1016/S0140-6736(08)61462-218942195

[20] Armand L. Les touristes français à l’étranger en 2006: résultats issus du suivi de la demande touristique (Direction du Tourisme). Bulletin Epidémiologique Hebdomadaire. 2007; 25–26: 218–21 [cited 2011 Aug 23]. http://www.invs.sante.fr/beh/2007/25_26/index.htm

[21] Bourhy H, Dacheux L, Strady C, Mailles A. Rabies in Europe in 2005. Euro Surveill. 2005;10:213–6.16371690

[22] Bourhy H, Kissi B, Audry L, Smreczak M, Sadkowska-Todys M, Kulonen K, Ecology and evolution of rabies virus in Europe. J Gen Virol. 1999;80:2545–57.1057314610.1099/0022-1317-80-10-2545

[23] Cliquet F, Aubert M. Elimination of terrestrial rabies in western European countries. Dev Biol (Basel). 2004;119:185–204.15747421

[24] Banyard AC, Hartley M, Fooks AR. Reassessing the risk from rabies: a continuing threat to the UK? Virus Res. 2010;152:79–84. Epub 2010 Jun 15. 10.1016/j.virusres.2010.06.00720558215PMC7126864

[25] Peigue-Lafeuille H, Bourhy H, Abiteboul D, Astoul J, Cliquet F, Gouda M, Human rabies in France in 2004: update and management [in French]. Med Mal Infect. 2004;34:551–60.1560393010.1016/j.medmal.2004.07.031

[26] Krause R, Bagó Z, Revilla-Fernández S, Loitsch A, Allerberger F, Kaufmann P, Travel-associated rabies in Austrian man. Emerg Infect Dis. 2005;11:719–21.1589012710.3201/eid1105.041289PMC3320390

[27] Schmiedel S, Panning M, Lohse A, Kreymann KG, Gerloff C, Burchard G, Case report on fatal human rabies infection in Hamburg, Germany, March 2007. Euro Surveill. 2007;12:E070531.5.10.2807/esw.12.22.03210-en17868587

[28] Johnson N, Brookes SM, Fooks AR, Ross RS. Review of human rabies cases in the UK and in Germany. Vet Rec. 2005;157:715. 10.1136/vr.157.22.71516311386

[29] Health Protection Agency. Case of rabies imported into the UK. Health protection report, 19 December 2008 [cited 2008 Dec 19]. http://www.hpa.org.uk/hpr/news/default.htm#rabies

[30] Höjer J, Sjöblom E, Berglund O, Hammarin AL, Grandien M. The first case of rabies in Sweden in 26 years. Inform travellers abroad about risks and treatment following suspected infection [in Swedish]. Lakartidningen. 2001;98:1216–20.11293124

[31] Rimhanen-Finne R, Järvinen A, Kuusi M, Quiambao BP, Malbas FF Jr, Huovilainen A, Imported human rabies, the Philippines and Finland, 2007. Emerg Infect Dis. 2010;16:1318–9. 10.3201/eid1608.09138020678336PMC3298315

[32] Gautret P, Adehossi E, Soula G, Soavi MJ, Delmont J, Rotivel Y, Rabies exposure in international travelers: do we miss the target? Int J Infect Dis. 2010;14:e243–6. Epub 2009 Aug 12. 10.1016/j.ijid.2009.05.00919674923

[33] Altmann M, Parola P, Delmont J, Brouqui P, Gautret P. Knowledge, attitudes, and practices of French travelers from Marseille regarding rabies risk and prevention. J Travel Med. 2009;16:107–11. 10.1111/j.1708-8305.2008.00283.x19335810

[34] Mailles A, Bourhy H, De Valk H, Dacheux L, Servas V, Capek I, Human and animal exposure to a rabid dog illegally imported into France, August 2004. Euro Surveill. 2004;8:1–3.

[35] Rooney R. A dog with rabies in Nîmes, France. Euro Surveill. 1998;2:pii=1175.

[36] Servas V, Mailles A, Neau D, Castor C, Manetti A, Fouquet E, An imported case of canine rabies in Aquitaine: investigation and management of the contacts at risk, August 2004–March 2005. Euro Surveill. 2005;10:222–5.16371687

[37] French multidisciplinary investigation team. Identification of a rabid dog in France illegally introduced from Morocco. Euro Surveill. 2008;13:pii:8066 [cited 2011 Aug 23]. http://www.eurosurveillance.org/viewarticle.aspx?articleid=806610.2807/ese.13.11.08066-en18768130

[38] Regulation of the European Parliament and of the Council of 26 May 2003 on the animal health requirements applicable to the non-commercial movement of pet animals and amending Council Directive 92/65/EEC. EC no. 998/2003 [cited 2011 Aug 23]. http://ec.europa.eu/food/animal/liveanimals/pets/annex2c_reg998_03_en.pdf

[39] Rabies vaccines. WHO position paper. Wkly Epidemiol Rec. 2007;82:425–35.18064757

[40] Gautret P, Shaw M, Gazin P, Soula G, Delmont J, Parola P, Rabies post-exposure prophylaxis in returned injured travellers from France, Australia and New Zealand: a retrospective study. J Travel Med. 2008;15:25–30. 10.1111/j.1708-8305.2007.00164.x18217866

# Review article: Risk for Rabies Importation from North Africa

**DOI:** 10.3201/eid1712.110300

**Authors:** Philippe Gautret, Florence Ribadeau-Dumas, Philippe Parola, Philippe Brouqui, Hervé Bourhy

**Affiliations:** Author affiliations: Hôpital Nord, Marseille, France (P. Gautret, P. Parola, P. Brouqui);; Institut Pasteur, Paris, France (F. Ribadeau-Dumas, H. Bourhy)

## Earning CME Credit

To obtain credit, you should first read the journal article. After reading the article, you should be able to answer the following, related, multiple-choice questions. To complete the questions (with a minimum 70% passing score) and earn continuing medical education (CME) credit, please go to www.medscape.org/journal/eid. Credit cannot be obtained for tests completed on paper, although you may use the worksheet below to keep a record of your answers. You must be a registered user on Medscape.org. If you are not registered on Medscape.org, please click on the New Users: Free Registration link on the left hand side of the website to register. Only one answer is correct for each question. Once you successfully answer all post-test questions you will be able to view and/or print your certificate. For questions regarding the content of this activity, contact the accredited provider, CME@medscape.net. For technical assistance, contact CME@webmd.net. American Medical Association’s Physician’s Recognition Award (AMA PRA) credits are accepted in the US as evidence of participation in CME activities. For further information on this award, please refer to http://www.ama-assn.org/ama/pub/category/2922.html. The AMA has determined that physicians not licensed in the US who participate in this CME activity are eligible for *AMA PRA Category 1 Credits*™. Through agreements that the AMA has made with agencies in some countries, AMA PRA credit may be acceptable as evidence of participation in CME activities. If you are not licensed in the US, please complete the questions online, print the certificate and present it to your national medical association for review.

## Article Title: Risk for Rabies Importation from North Africa

## CME Questions

1. What is the most common animal vector associated with fatal cases of rabies?A. BatB. CatC. SquirrelD. Dog2. Which of the following statements regarding efforts to prevent rabies in North Africa is most accurate?A. Greatest access to rabies postexposure prophylaxis (PEP) is in SudanB. Least access to rabies PEP is in TunisiaC. Mass vaccination campaigns and the elimination of free-roaming dogs have been somewhat effective in TunisiaD. The elimination of free-roaming dogs has been largely effective in Egypt3. Which of the following statements regarding the management of rabies cases in France is most accurate?A. Most patients in ARMC were exposed to animals outside of FranceB. The use of rabies PEP for injuries incurred in other countries increased between 1996 and 2009C. Sudan is the country at highest risk for rabies exposure requiring rabies PEPD. The use of rabies PEP is similar regardless of injury in North Africa vs. other international locations4. Which of the following groups of patients should be most strongly considered for rabies preexposure vaccination prior to travel to North Africa?A. Tourists visiting for a limited timeB. Older adultsC. Visitors to countries with the mouse brain rabies vaccineD. Women

## Activity Evaluation

**Table:** 

1. The activity supported the learning objectives.				
Strongly Disagree				Strongly Agree
1	2	3	4	5
2. The material was organized clearly for learning to occur.				
Strongly Disagree				Strongly Agree
1	2	3	4	5
3. The content learned from this activity will impact my practice.				
Strongly Disagree				Strongly Agree
1	2	3	4	5
4. The activity was presented objectively and free of commercial bias.				
Strongly Disagree				Strongly Agree
1	2	3	4	5
